# 3-(((1*S*,3*S*)-3-((*R*)-Hydroxy(4-(trifluoromethyl)phenyl)methyl)-4-oxocyclohexyl)methyl)pentane-2,4-dione: Design and Synthesis of New Stereopure Multi-Target Antidiabetic Agent

**DOI:** 10.3390/molecules27103265

**Published:** 2022-05-19

**Authors:** Abdul Sadiq, Mater H. Mahnashi, Umer Rashid, Muhammad Saeed Jan, Mohammed Abdulrahman Alshahrani, Mohammed A. Huneif

**Affiliations:** 1 Department of Pharmacy, Faculty of Biological Sciences, University of Malakand, Chakdara 18000, Dir (L), KP, Pakistan; 2 Department of Pharmaceutical Chemistry, College of Pharmacy, Najran University, Najran 55461, Saudi Arabia; 3 Department of Chemistry, COMSATS University Islamabad, Abbottabad Campus, Abbottabad 22060, KP, Pakistan; umerrashid@cuiatd.edu.pk; 4 Department of Pharmacy, University of Swabi, Swabi 23561, KP, Pakistan; saeedjanpharmacist@gmail.com; 5 Department of Clinical Laboratory Sciences, Faculty of Applied Medical Sciences, Najran University, P.O. Box 1988, Najran 61441, Saudi Arabia; maalshahrani@nu.edu.sa; 6 Pediatric Department, Medical College, Najran University, Najran 55461, Saudi Arabia; huneif@hotmail.com

**Keywords:** chiral synthesis, antidiabetic, molecular docking, α-glucosidase, α-amylase, PTP1B and DPPH

## Abstract

The chiral drug candidates have more effective binding affinities for their specific protein or receptor site for the onset of pharmacological action. Achieving all carbon stereopure compounds is not trivial in chemical synthesis. However, with the development of asymmetric organocatalysis, the synthesis of certain vital chiral drug candidates is now possible. In this research, we have synthesized 3-(((1*S*,3*S*)-3-((*R*)-hydroxy(4-(trifluoromethyl)phenyl)methyl)-4-oxocyclohexyl)methyl)pentane-2,4-dione (*S*,*S*,*R*-**5**) and have evaluated it potential as multi-target antidiabetic agent. The stereopure compound *S*,*S*,*R*-**5** was synthesized with a 99:1 enantiomeric ratio. The synthesized compound gave encouraging results against all in vitro antidiabetic targets, exhibiting IC_50_ values of 6.28, 4.58, 0.91, and 2.36 in α-glucosidase, α-amylase, PTP1B, and DPPH targets, respectively. The molecular docking shows the binding of the compound in homology models of the respective enzymes. In conclusion, we have synthesized a new chiral molecule (*S*,*S*,*R*-**5**). The compound proved to be a potential inhibitor of the tested antidiabetic targets. With the observed results and molecular docking, it is evident that *S*,*S*,*R*-**5** is a potential multitarget antidiabetic agent. Our study laid the baseline for the animal-based studies of this compound in antidiabetic confirmation.

## 1. Introduction

Chiral molecules are considered more suitable for a target protein or receptor within the living system [1]. In the overall pharmacological diversity of different target proteins/receptors, the stereoselectivity of a target cannot be overruled [2]. The normal enantioselective drugs result in diastereomeric properties when they bind themselves to a stereoselective site [3]. Within the body, the binding receptor is most often more favorable for one enantiomer than the other [4]. Despite the importance of chiral drugs and their interactions in three-dimensional, complex structures, many drugs are available in the market in the racemic form [5]. The economy might be one of the major causes for the existence of achiral drug molecules on the market. The concept of chirality was introduced by Louis Pasteur in the nineteenth century [6]. At the initial time, a major challenge was the chiral resolution of enantiomers. However, with the passage of time and research, new methods were introduced for the synthesis of chiral molecules. Asymmetric organocatalysis sets among the major approaches for chiral synthesis [7]. Though the concept of organocatalysis was initially reported in 1971 [8]. However, due to several limitations, the methods were not expanded. At the start of the current century, it was Binjamin List who introduced and then developed the concept of organocatalysis [9]. Binjamin List and David W.C. MacMillan were awarded the Noble Prize in chemistry this year for the development of asymmetric organocatalysis. Over the past twenty years, asymmetric organocatalysis has been one of the vital tools for the synthesis of various chiral molecules [10]. Both covalent-based bifunctional and non-covalent organocatalysts have been employed for asymmetric Michael, aldol, Mannich, Diels-Alder, and other reactions [11,12,13].

A Me-too drug concept or racemic switch is one of the easiest approaches for the development of a newly marketed drug with greater efficacy [14]. Among the chiral drugs, major pharmacological targets are diabetes, CVS, neurological disorders, cancer, analgesia, inflammation, etc. In our previous research, we have synthesized various chiral molecules that can have potentially drug-like properties [15,16,17,18]. Furthermore, we have also elaborated on the binding of a chiral drug to a target protein for the management of analgesia and inflammation [18,19]. Molecular docking is one of the dry-laboratory approaches for the confirmation of binding interactions of a drug molecule within the binding protein [20,21,22]. Molecular docking is also capable of providing information prior to the in vitro testing of a compound [23,24,25]. Herein, we have designed and synthesized a new enantio- and diastereopure *S*,*S*,*R*-**5** with a site-specific asymmetric organocatalytic approach. With preliminary molecular docking studies on various target proteins, we were convinced that the synthesized compound is most suitable for the antidiabetic target protein. Based on the above conceptualization, we have explored the multitarget in vitro antidiabetic potential.

**Figure 1:**
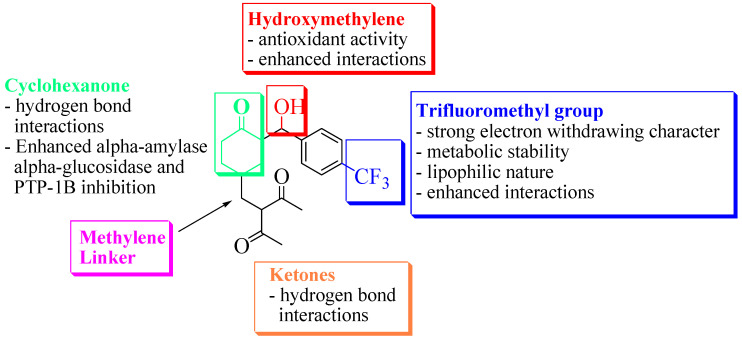
Design rational for current research.

In the drug design chemistry and development of bioactive therapeutics, the use of the trifluoromethyl group has been increased due to its strong electron-withdrawing character and lipophilicity. A number of FDA-approved (e.g., sitagliptin) and reported investigational drugs to contain this group [26,27]. Hence, we incorporated the CF_3_ group into our designed triketone. To interact with the amino acid residues, the presence of carbonyl oxygen and a hydroxy methyl group is essential. Moreover, hydroxy-containing molecules were also reported to have enhanced antioxidant activity. Our rationale for the design of the target compound is shown in Figure 1.

## 2. Results

### 2.1. Chemistry of Stereopure 3-(((1S,3S)-3-((R)-hydroxy(4-(trifluoromethyl)phenyl)methyl)-4-oxocyclohexyl)methyl)pentane-2,4-dione

**Scheme 1:**
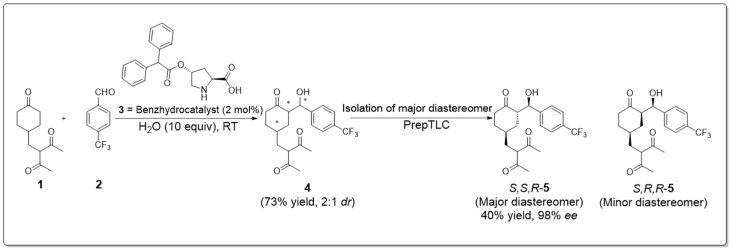
Synthetic approach to *S*,*S*,*R*-**5**.

The asymmetric organocatalysis is now well-established for the synthesis of various asymmetric products. Different types of organocatalysts have been employed to date for various chiral syntheses. The nucleophilic part of a bifunctional organocatalyst normally targets an active part of the donor compound. However, it is very rare that an organocatalyst selectively forms the nucleophilic enamine at one carbonyl while leaving the other on the same molecule unreactive. This type of aldol reaction has been recently reported by Nugent et al. [28]. Using the same site-selective approach, we have reacted a triketone (**1**) with 4-CF_3_-benzaldehyde (**2**) in the presence of a chiral organocatalyst (**3**) in the presence of water. The diastereomeric ratio was confirmed by the crude 1H NMR and was found to be 2:1. Compound **4** was initially purified as a mixture of diastereomers with an isolated yield of 73%. The diastereomeric mixture was then loaded onto a pre-coated silica gel preparative TLC plate as a streak. The separation proceeded, and we were able to isolate a single major diastereomer of *S*,*S*,*R*-**5** as shown in Scheme 1. The observed enantiomeric excess (*ee*) for the compound *S*,*S*,*R*-**5** was 98%, as confirmed by the chiral HPLC analysis.

All the analytical details related to Scheme 1 are provided in the Supplementary Materials (Figures S1–S10).

To confirm the stereochemistry of the compound, chiral HPLC with a chiral OD-H column was used. The solvents used were *n*-heptane and isopropanol. The ratios of the solvents were 90:10 for the *n*-heptane and isopropanol, respectively. The flow rate in the column was 0.6 mL/min. The compound *S*,*S*,*R*-**5** was also synthesized in a racemic form with an achiral organocatalyst. The enantiomers of compound *S*,*S*,*R*-**5** were separated on chiral HPLC as a 1:1 mixture. Then the chiral HPLC of the stereopure compound was performed and compared with the HPLC data of the racemic compound. The retention times of the enantiomers’ peaks from the racemic were matched with the peaks of the chiral compounds. The *ee* was calculated from the percent area of the major and minor enantiomers.

The racemic-**5** was also prepared, and the two enantiomers were separated by chiral HPLC analysis. The two enantiomers gave peaks between 15.763 and 18.917 min retention times (Figure S9, Supplementary Materials). The chiral HPLC analysis of the stereopure compound *S*,*S*,*R*-**5** confirmed that the major enantiomer is the one eluting later in the same method, i.e., at 18.928 min with a percent area of ~99% as shown in Figure S10 of the Supplementary Materials. The chiral HPLC pattern of the compound was compared with the published literature having a similar nucleus and the stereochemical assignments were given according to the published literature [28].

### 2.2. In-Vitro Multitarget Antidiabetic Results

Initially, we performed the comparative screening of major (*S*,*S*,*R*-**5**), minor (*S*,*S*,*R*-**5**), and racemic (*Rac*-**5**) analogues for various in vitro targets, as shown in Table 1. The purpose was to check the possible potencies of these stereoisomers and their corresponding racemic analogues in the in vitro targets. Therefore, we only used single concentrations of all the compounds, i.e., 500 micromoles/mL. The major diastereomer (*S*,*S*,*R*-**5**) exhibited 83.13, 78.85, 88.35, and 92.23% inhibitions in α-glucosidase, α-amylase, PTP-1B, and DPPH assays. In comparison, the minor diastereomer (*S*,*S*,*R*-**5**) was comparatively lower in activity, giving percent inhibitions of 73.15, 72.65, 65.42, and 70.21 in the tested in vitro assays. We also tested the potency of the racemic analogue (*Rac*-**5**). The Rac-D was very poor in activity, as shown in Table 1.

Based on the preliminary screening shown in Table 1, we further extended only the major compound for all the activities. The compound *S*,*S*,*R*-**5** in stereopure form (single diastereomer, 99:1 enantiomeric ratio (*er*)) was subjected to various in vitro antidiabetic enzymes, as shown below.

The alpha-glucosidase inhibitory results of compound *S*,*S*,*R*-**5,** are shown in Table 2. In the alpha-glucosidase inhibition assay, the compound at 500 μM/mL exhibited 83.13% inhibition. Similarly, a concentration-dependent response in activity was observed at 250, 125, 62.5, and 31.25 μM/mL. The observed IC_50_ value for compound *S*,*S*,*R*-**5** was 6.28 μM. In comparison to our stereopure compound, the standard acarbose exhibited an IC_50_ value of 2.0 μM.

In almost a similar pattern to that of glucosidase, our stereopure compound *S*,*S*,*R*-**5** showed encouraging alpha-amylase inhibitory activity as shown in Table 3. The observed percent inhibitions were 78.85, 73.08, 68.90, 62.28, and 58.47% at concentrations of 500, 250, 125, 62.5, and 31.25 μM/mL, respectively. The observed IC_50_ value for compound *S*,*S*,*R*-**5** was 4.58 μM in comparison to the standard acarbose (IC_50_ value 1.58 μM).

In the protein tyrosine phosphatase 1B (PTP-1B) assay, our stereopure compound *S*,*S*,*R*-**5** excelled in antidiabetic activity to the standard ursolic acid, as shown in Table 4. The compound *S*,*S*,*R*-**5** at 500, 250, 125, 62.5, and 31.25 μM/mL exhibited percent inhibitions of 88.35, 84.36, 79.62, 76.16, and 73.67%, respectively. The IC_50_ value in the PTP-1B assay was 0.91 μM in comparison to the standard ursolic acid value, which was 1.35 μM.

The free radicals within the body are implicated in the onset of various diseases of neurological origin, inflammation, and diabetes. The excessive free radicals within the body cause lipid peroxidation, non-enzymatic proteins glycation, and oxidation of glucose, which ultimately complicates diabetes mellitus. So, in this continuation, we supplemented the multitarget antidiabetic potency of compound *S*,*S*,*R*-**5** with the antioxidant assay. For the antioxidant assay, we used DPPH free radicals’ inhibitions, as shown in Table 5. In the antioxidant assay, our stereopure compound exhibited excellent supplemented results with an IC_50_ value of 2.36 μM. The antioxidant potential of our compound was compared with that of positive control ascorbic acid, which gave an IC_50_ value of 0.85 μM.

We also tested the toxicity profile of our compound *S*,*S*,*R*-**5** by using the simple acute toxicity test in experimental albino mice. The mice were acquired and used in the animal house of our university as per the standard guidelines of the departmental ethical committee. As per the standard procedure, we gave various concentrations of compound *S*,*S*,*R*-**5** to albino mice and observed them for the next 72 h. After 72 h, we observed no unwanted behavioral changes or lethality.

### 2.3. Molecular Docking Studies

**Figure 2:**
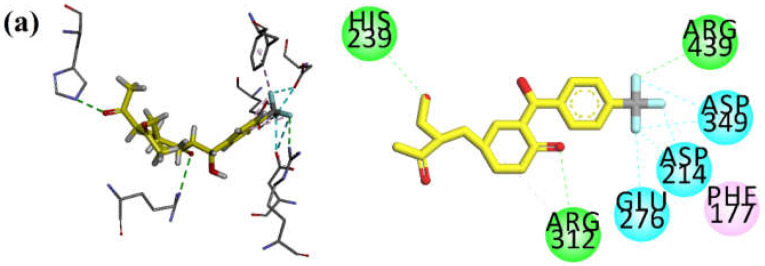

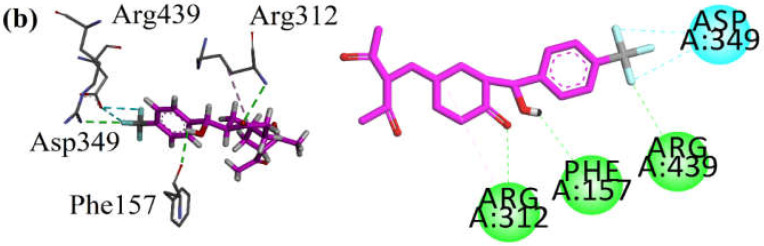
3-D/2-D interaction plot of (**a**) major (*S*,*S*,*R*-**5**, as yellow stick model) and; (**b**) minor (*S*,*S*,*R*-**5**, as pink stick model) diastereoisomers into the binding site of homology modeled α-glucosidase.

**Figure 3:**
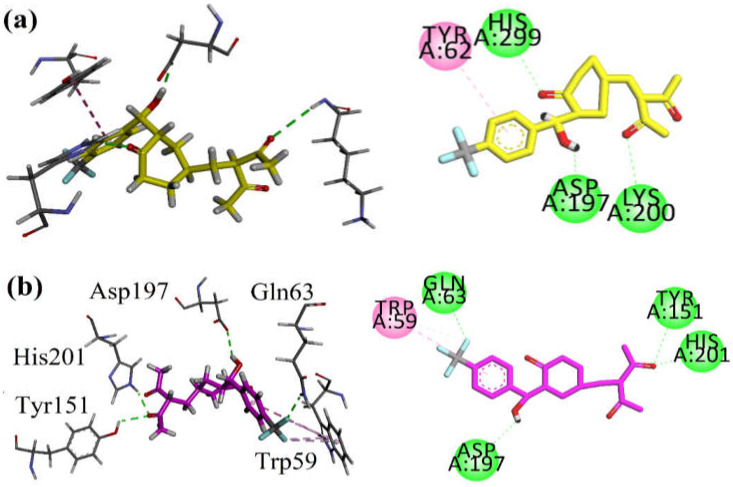
3-D/2-D interaction plot of (**a**) major (*S*,*S*,*R*-**5**, as yellow stick model) and; (**b**) minor (*S*,*S*,*R*-**5**, as pink stick model) diastereosomers into the binding site of homology modeled α-amylase.

**Figure 4:**
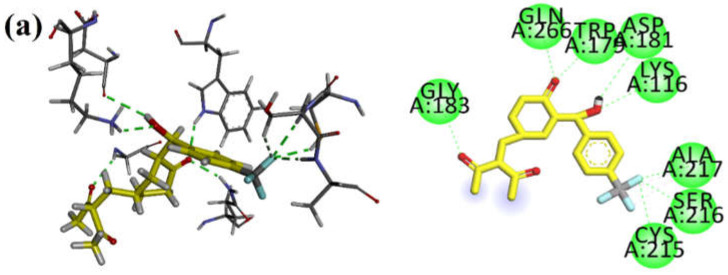

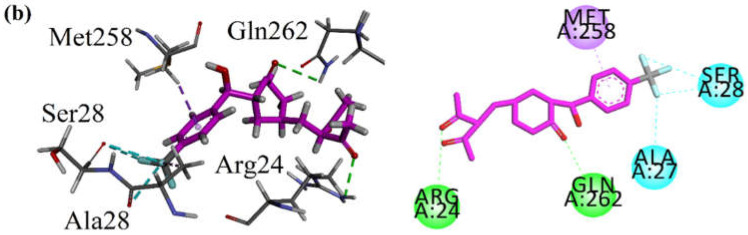
3-D/2-D interaction plot of (**a**) major (*S*,*S*,*R*-**5**, as yellow stick model) and; (**b**) minor (*S*,*S*,*R*-**5**, as pink stick model) diastereosomers into the binding site of homology modeled PTP-1B.

The synthesized triketone was docked into the binding sites of α-Glucosidase, α-Amylase, and PTP-1B. The predictive power of docking was used to explore the binding orientation and binding energy changes in the major (*S*,*S*,*R*-**5**) and minor (*S*,*S*,*R*-**5**) diastereoisomers. For this purpose, the homology modeled structure of α-glucosidase previously reported by our research group was used for docking [29]. While 3-D crystal structures of α-amylase and PTP-1B were obtained from PDB with accession numbers 4W93 and 1NNY, respectively. The interaction plots of the major (*S*,*S*,*R*-**5**) and minor (*S*,*S*,*R*-**5**) diastereoisomers are shown in Figure 2, Figure 3 and Figure 4. Major diastereoisomer (*S*,*S*,*R*-**5**, forms three hydrogen bond interactions with His239, Arg312, and Arg439. Apart from hydrogen bond interactions, the ligand enzyme complex of *S*,*S*,*R*-**5** is also stabilized by forming halogen interactions with catalytic triad residues Asp214 and Glu276 (Figure 2a). While minor diastereoisomer (*S*,*S*,*R*-**5**) oriented towards Phe157, Arg439, and Arg312 and forms hydrogen bond interactions in the binding site of homology modeled α-glucosidase. While Asp349 forms interactions with fluorine atoms (Figure 2b). The computed binding energy values for major and minor diastereoisomers are −7.7709 kcal/mol and −6.3521 kcal/mol, respectively.

For α-Amylase, major diastereoisomer forms π-π interactions with Tyr62 and hydrogen bond interactions with Asp197, Lys200, and His299 (Figure 3a). Minor diastereoisomer forms four hydrogen bond interactions with Gln63, Tyr151, Asp197, and His201 (Figure 3b). The computed binding energy values for major and minor diastereosomers are −6.7498 kcal/mol and −6.1939 kcal/mol, respectively. For PTP-1B, the major diastereoisomer forms hydrogen bond interactions with Lys116, Trp179, Asp181, Gly183, Cys215, Ser216, Ala217, and Gln266 (Figure 4a). While minor diastereosomers π-σ interactions with Met258. Two hydrogen bond interactions with Arg24 and Gln262. While Ala2 and Ser28 form interactions with fluorine atoms (Figure 4b). The computed binding energy values for major and minor diastereoisomers are −6.6024 kcal/mol and −4.7403 kcal/mol, respectively.

## 3. Discussion

Diabetes is a chronic disorder of the current era and is mainly associated with lifestyle [30]. In this disorder, the blood glucose level increases from the normal level, causing the condition called hyperglycemia [31]. A persistently high blood glucose level affects the vital organs and functions of the body, such as the heart, kidneys, and eyes, and may cause many other severe conditions [32]. The two major types of diabetes, i.e., type 1 and 2, are mainly different in terms of insulin level and availability within the body [33]. Type 1 patients are unable to have the required level of insulin necessary for their body [34]. So, the only remedy for type 1 patients is the injection of insulin on a regular basis. On the other hand, in type 2 patients, there is insufficient action from insulin or resistance to action [35]. Type 2 has several remedies, such as insulin or other medications [36]. Moreover, this type can also be controlled by changing one’s lifestyle. However, due to the limitations associated with current drugs, medicinal chemists are in constant research for modifying the existing drug or the discovery of new drug molecules [37,38].

In this research, we have designed and synthesized a new compound for the possible management of diabetic targets. We have synthesized the compounds in three different forms, i.e., the major diastereomer, minor diastereomer, and their corresponding racemic analogue. We initially tested all three analogues and observed that the major diasteremer was potent in giving better percent inhibitions, as shown in Table 1. So, based on this initial screening, we extended the major diastereomer for further activities using different concentrations. Our designed compound exhibited percent alpha-glucosidase inhibitions of 83.13, 78.83, 72.70, 66.43, and 61.06 at concentrations of 500, 250, 125, 62.5, and 31.25 μM/mL. The calculated IC_50_ value of our compound was 6.28 μM, which was close enough to the standard acarbose (IC_50_ of 2.00 μM), as shown in Table 2. Similarly, our compound was also observed to be potent, with an IC_50_ value of 4.58 μM in alpha-amylase activity, as shown in Table 3. In the alpha-amylase assay, the IC_50_ of the standard acarbose was 1.58 μM. With our major diasteromer, we observed an outstanding result in the PTP-1B assay, as shown in Table 4. In this assay, our compound excelled at the activity of the standard drug ursolic acid. The observed IC_50_ value of our compound was 0.91 μM in comparison to the standard drug IC_50_, which was 1.35 μM. It is obvious that the antioxidants protect the beta cells, which are mainly responsible for the production of insulin. So, we also performed the DPPH antioxidant assay as a supplementary target. In the DPPH assay, our major diastereomer exhibited an IC_50_ of 2.36 μM in comparison to ascorbic acid (IC_50_ of 0.85 μM), as shown in Table 5.

The medicinal chemists used two major approaches for the discovery of new potential drugs, i.e., natural or synthetic approaches [39,40,41,42]. Natural products are one of the safest sources for effective drug discovery [43,44]. However, the major disadvantages associated with a natural product are the tedious process and reproducibility [45]. Therefore, medicinal chemists are also modifying existing drugs or synthesizing new drug molecules from commercially available chemicals [46]. The current study is a vital step for the discovery and development of new drug candidates for the management of diabetes.

## 4. Materials and Methods

### 4.1. Chemistry

#### 4.1.1. Setting up of Chemical Reaction

To a 2.0 mL reaction vial was added 1.625 mg (0.005 equiv, 2 mol%) of **3** followed by 78.85 mg (1.5 equiv, 0.375 mmol) between **1** and 45.05 µL (10.0 equiv, 2.5 mmol) of water. Then 43.53 mg (1.0 equiv, 0.25 mmol) of freshly purified **2** was added to it carefully and continued gentle stirring. The progress of this reaction was monitored by TLC and NMR. The reaction proceeds very fast in first from 5 to 10 h and reaches from 50 to 70% (Depending upon the care taken in reaction procedure). Afterward, the reaction slows down and after 20 h, no obvious change can be observed in the starting material consumption. After 20 h, the reaction mixture was diluted with 1 mL of dichloromethane and stirred for 2 min to dissolve the reaction mixture. The reaction mixture was transferred into a 25 mL separating funnel and was added 5 mL of each water and DCM. The organic layer was separated, and the aqueous layer was extracted two more times with 5 mL of DCM to remove all the crude product. The DCM layers were combined and dried with sodium sulfate. The sodium sulfate was removed by filtering it and was then dried by high vacuum using rotary evaporator up to 40 °C. The crude product, conversion, and *dr* were confirmed from the ^1^H NMR spectrum of the crude product.

#### 4.1.2. Purification of Compound (Diastereomeric Mixture)

For the isolation of the aldol product **4** (both diastereomers), column was packed with silica and slowly eluted with petroleum ether and ethyl acetate. The starting eluting ratio of the solvent was 98:2 (pet ether and ethyl acetate). Small fractions of not more than 20–25 mL were collected. The observed *R*_f_ value for the aldol product ranged from 0.27 to 0.33 (for both overlapping diastereomers in petroleum ether and acetone with 9:1 ratio). Due to overlapping, it was not practically possible to isolate/separate the two diastereomers by normal column chromatography. The aldol product (mixture of diastereomers) was purified by column chromatography. The observed isolated yield for a 0.25 mmol reaction was 73% (70 mg of the semisolid material).

#### 4.1.3. Isolation of Single Diastereomer

The mixture of diastereomers 70 mg (73%) was loaded on a preparative TLC and was developed with petroleum ether and ethyl acetate (9:1). The portion of the developed TLC, which only contained the major diastereomer (*R*_f_ = 0.28) was scratched with a cleaned spatula. The major diastereomer was dissolved out with acetone and was filtered to remove silica gel. The compound appeared as off-white color semi-solid. The product was dried and was confirmed by NMR and MS.

In ^1^H NMR spectrum of major diastereomer of compound *S*,*S*,*R*-**5**, two doublets appeared between 7.55 and 7.34 chemical shifts, respectively, with coupling constant (*J*) value of 7.75 Hz. These two doublets represent the aromatic part of the compound. The proton at alpha position to the OH group gave doublet at 5.38 with *J* value of 7.05. The proton between the two carbonyls appeared as a triplet at 3.54 with *J* value of 7.04. The broad singlet for the OH group appeared at 2.90. The methyl groups appeared at between 2.03 and 2.01 with unequal integral values due to tautomerism. All the remaining protons (10 aliphatic Hs) appeared multiplets between 2.34 and 1.31. The ^13^C NMR was also clear showing signals at 213.50, 213.31, 203.78, 203.73, 191.59, 145.31, 126.16, 126.09, 125.38, 125.34, 122.88, 108.44, 99.99, 70.10, 69.91, 66.76, 55.70, 55.59, 41.63, 41.32, 38.07, 34.47, 33.82, 33.75, 33.13, 32.81, 31.98, 31.71, 29.00, 28.99, and 23.49. The MS and HRMS spectra [M − 1]^+^ were in accordance with the calculated molecular weight (384.1548) of our synthesized compound (C_20_H_23_F_3_O_4_), giving between 383 and 383.1688 m/z, respectively. The distinct bands in IR spectrum were noted as 3444, 2930, 2861, 1699, 1619, 1419, 1359, 1324, 1161, 1120, 1066, 1016, 961, 925, 847, 751, 703, 659, and 609 cm^−1^.

#### 4.1.4. Synthesis of Racemic Compound

To a 2.0 mL reaction vial was added racemic proline (5 mol%) followed by 1.5 equiv of between **1** and 10.0 equiv of water. Then 1.0 equiv of freshly purified **2** was added to it carefully and continued gentle stirring. The progress of this reaction was monitored by TLC. After the product formation, the targeted compound was purified by column chromatography and the structure was confirmed with 1H NMR analysis. The racemic stereoisomer was separated on chiral HPLC analysis for comparison with the chiral compound.

#### 4.1.5. HPLC Analysis/Stereochemistry of Compound *S*,*S*,*R*-**5**

To confirm the stereochemistry of compound, chiral HPLC with chiral OD-H column was used. The solvents used were *n*-heptane and isopropanol. The ratios of the solvents were 90:10 for the *n*-heptane and isopropanol, respectively. The flow rate in the column was 0.6 mL/min. The compound *S*,*S*,*R*-**5** was also synthesized in a racemic form with achiral organocatalyst. The enantiomers of compound *S*,*S*,*R*-**5** were separated on chiral HPLC as 1:1 mixture. Then the chiral HPLC of the stereopure compound was performed and compared with the HPLC data of racemic compound. The retention times of the enantiomers’ peaks from the racemic were matched with the peaks of the chiral compounds. The enantiomeric excess (*ee*) was calculated from the percent area of the major and minor enantiomers. We also confirmed the optical rotation value of compound *S*,*S*,*R*-**5** as being ${\left[a\right]}_{D}^{20}$ + 83.5 (CHCl_3_).

### 4.2. In-Vitro Assays

#### 4.2.1. In-Vitro α-Glucosidase Inhibition

The *α*-glucosidase inhibitory activity on the *S*,*S*,*R*-**5** was performed as per the reported protocols [47]. In this assay, the standard acarbose was used in parallel to the compound. The *α*-glucosidase solution (0.5 units/mL) was prepared in phosphate buffer (0.1 M, 6.90 pH). *p*-Nitrophenyl-*α*-d-glucopyranoside (5 mM) was added as substrate solution to the buffer solution. Different concentrations of the compound, i.e., 500, 250, 125, 62.5, and 31.25 μM were prepared. The enzyme solutions were mixed with the compound’s solution and were incubated for 15 min at 37 °C. To these solutions were added the pre-prepared substrate solution and the repetition of incubation. The final reaction was completed with the addition of sodium carbonate solution (0.2 M, 80 μL). The combined solution excluding the *α*-glucosidase was used as a blank solution. The absorbances of the solutions were recorded at 405 nm. The observations were repeated three times. The percent inhibitions at each concentration and the IC_50_ values were calculated according to the standard formula [48].

#### 4.2.2. In-vitro α-Amylase Inhibition

The potential of stereopure *S*,*S*,*R*-**5** against the *α*-amylase was also determined as per the protocols [49]. The enzyme solution was prepared in the phosphate buffer and was mixed with different concentrations of the compound to allow the reaction. All the solutions were incubated as per the method. Dinitrosalicylic acid was added to the compound and standard solutions. The respective solutions were heated in water baths for a few minutes. The absorbances of the solutions were recorded at 656 nm. The observations were repeated three times. The percent inhibitions at each concentration and the IC_50_ values were calculated according to the standard formula [50].

#### 4.2.3. Protein Tyrosine Phosphatase 1B Inhibition

The third in vitro antidiabetic target studied on the stereopure compound *S*,*S*,*R*-**5** was protein tyrosine phosphatase 1B as per the standard procedure [49]. A buffer solution with pH 7 of 3,3-dimethyl glutarate was used. The solutions of protein tyrosine phosphatase 1B (10 mM), 4-nitrophenolphosphate (1 mM), and various concentrations of stereopure compound *S*,*S*,*R*-**5** were prepared. All the solutions were incubated at 27 °C for 40 min. The absorbances of the solutions were recorded at 405 nm. The observations were repeated three times. The percent inhibitions at each concentration and the IC_50_ values were calculated according to the standard formula.

#### 4.2.4. DPPH Free Radical Scavenging Assay (Antioxidant Assay)

To supplement the role of free radicals in diabetes, the free radical scavenging potential of compound *S*,*S*,*R*-**5** was also determined as per the standard method [51]. Different concentrations of the stereopure compound were prepared and DPPH solution in methanol (0.004%) were added to it. The solutions were incubated for half an hour. The absorbances of the solutions were recorded at 517 nm. The observations were repeated three times. The percent inhibitions at each concentration and the IC_50_ values were calculated according to the standard formula [52].

### 4.3. Statistical Analysis

The statistical analyses in all in vitro assays were performed as per our reported methods [53].

### 4.4. Molecular Docking

Both of the synthesized diastereomers, i.e., *S*,*S*,*R*-**5** and *S*,*S*,*R*-**5** were tested in silico for their binding orientations and interaction patterns. Three-dimensional (3-D) crystal structures of the target enzymes were obtained from Protein Data Bank (PDB). The accession codes for the obtained enzymes are 4W93 and 1NNY for α-amylase and PTP-1B, respectively [54,55,56]. While previously constructed homology model of α-glucosidase was used for docking studies. Redock method of docking protocol validation was used and comparison of binding orientation between redocked conformation of native ligands and experimental ligands was made. A 3-D preparation of ligand and downloaded enzymes was carried out using our previous reported procedures [57]. Docking was carried out using validated protocol. Analysis of docking results was carried out by the analysis of 3-D interaction plots by using discovery studio visualizer.

## 5. Conclusions

In conclusion, we can claim that we have synthesized *S*,*S*,*R*-**5** for the first time in stereopure form. The chiral compound (99:1 enantiomeric ratio) proved to be a potential inhibitor of various in vitro antidiabetic targets. Overall, the compound showed a parallel activity profile to the respective standard drugs used. The molecular docking studies also supported our results. Our results show that this new compound would be worth evaluating for in vivo antidiabetic studies.

## 6. Patents

We would like to publish a patent specifically on the chemistry portion of our manuscript.

## Figures and Tables

**Table 1:** Initial screening of major (*S*,*S*,*R*-**5**), minor (*S*,*S*,*R*-**5**) and racemic (*Rac*-**5**) for various in vitro targets.

Compound (500 μM/mL)	α-Glucosidase % Inhibition	α-Amylase % Inhibition	PTP-1B % Inhibition	DPPH % Inhibition
*S*,*S*,*R*-**5**	83.13 ± 0.80	78.85 ± 2.24	88.35 ± 0.89	92.23 ± 0.22
*S*,*S*,*R*-**5**	73.15 ± 1.23	72.65 ± 0.65	65.42 ± 1.02	70.21 ± 2.11
*Rac*-**5**	62.31 ± 0.66	56.32 ± 1.66	54.00 ± 0.54	49.25 ± 1.05

**Table 2:** Alpha-glucosidase inhibitory results of *S*,*S*,*R*-**5**.

Samples	Mol wt.	Conc (μM/mL)	Percent Inhibition	IC_50_ (μM)
*S*,*S*,*R*-**5**	384	500 250 125 62.5 31.25	83.13 ± 0.80 *** 78.83 ± 0.73 *** 72.70 ± 0.51 *** 66.43 ± 0.70 *** 61.06 ± 0.70 ***	6.28 ± 0.10
Acarbose	645	500 250 125 62.5 31.25	95.20 ± 0.15 91.17 ± 0.53 86.98 ± 0.85 81.20 ± 0.65 77.80 ± 0.37	2.0 ± 0.06

Data was expressed as mean percent inhibition ± SEM. Two-way ANOVA followed by Bonferroni’s posttest was used for statistical analysis. *** *p* < 0.001, compared to that of the standard drugs.

**Table 3:** Alpha amylase inhibitory results of *S*,*S*,*R*-**5**.

Samples	Mol wt.	Conc (μM/mL)	Percent Inhibition	IC_50_ (μM)
*S*,*S*,*R*-**5**	384	500 250 125 62.5 31.25	78.85 ± 2.24 *** 73.08 ± 0.47 *** 68.90 ± 0.96 *** 62.28 ± 0.57 *** 58.47 ± 0.56 ***	4.58 ± 0.14
Acarbose	645	500 250 125 62.5 31.25	88.35 ± 0.89 84.36 ± 1.15 79.62 ± 0.03 76.16 ± 0.12 73.67 ± 0.35	1.58 ± 0.12

Data was expressed as mean percent inhibition ± SEM. Two-way ANOVA followed by Bonferroni’s posttest was used for statistical analysis. *** *p* < 0.001, compared to that of the standard drugs.

**Table 4:** Protein tyrosine phosphatase 1B inhibitory results of *S*,*S*,*R*-**5**.

Samples	Mol wt.	Conc (μM/mL)	Percent Inhibition	IC_50_ (μM)
*S*,*S*,*R*-**5**	384	500 250 125 62.5 31.25	88.35 ± 0.89 ^ns^ 84.36 ± 1.15 ^ns^ 79.62 ± 0.03 ^ns^ 76.16 ± 0.12 ^ns^ 73.67 ± 0.35 ^ns^	0.91 ± 0.10
Ursolic acid	457	500 250 125 62.5 31.25	90.83 ± 0.47 87.23 ± 0.96 82.29 ± 0.57 78.33 ± 0.55 76.03 ± 0.77	1.35 ± 0.13

Data was expressed as mean percent inhibition ± SEM. Two-way ANOVA followed by Bonferroni’s posttest was used for statistical analysis. ^ns^; not significant compared to that of the standard drugs.

**Table 5:** DPPH results of *S*,*S*,*R*-**5**.

Samples	Mol wt.	Conc (μM/mL)	Percent Inhibition	IC_50_ (μM)
*S*,*S*,*R*-**5**	384	500 250 125 62.5 31.25	92.23 ± 0.22 ^ns^ 87.45 ± 0.90 ^ns^ 81.90 ± 0.60 * 76.00 ± 0.30 ** 71.90 ± 0.45 ***	2.36 ± 0.10
Ascorbic acid	176	500 250 125 62.5 31.25	95.20 ± 0.15 91.17 ± 0.53 86.98 ± 0.85 81.20 ± 0.65 77.80 ± 0.37	0.85 ± 0.08

All values are taken as Mean ± SEM (*n* = 3). Two-Way ANOVA followed by Bonferoni test were followed, Values significantly different in comparison to standard drug, i.e., ^ns^; not significant *; *p* < 0.05, **; *p* < 0.01 and ***; *p* < 0.001.

## Data Availability

The whole data are available within the manuscript and Supplementary Materials.

## Supplementary Materials

The following are available online at https://www.mdpi.com/article/10.3390/molecules27103265/s1. The spectral information and chromatograms related to the compounds are provided in the supporting information. Figure S1: ^1^H NMR of the purified compound (mixture of diastereomers); Figure S2: ^13^C NMR of the purified compound (mixture of diastereomers); Figure S3: ^1^H NMR of the stereopure compound *S*,*S*,*R*-**5**; Figure S4: Expansions of the stereopure 1H NMR of compound *S*,*S*,*R*-**5**; Figure S5: ^13^C NMR of the stereopure compound *S*,*S*,*R*-**5**; Figure S6: HRMS of the stereopure compound *S*,*S*,*R*-**5**; Figure S7: MS data of the stereopure compound *S*,*S*,*R*-**5**; Figure S8: FT-IR analysis of the compound *S*,*S*,*R*-**5**; Figure S9: Chiral HPLC analysis of the racemic compound *Rac*-**5**; Figure S10: Chiral HPLC analysis of the stereopure compound *S*,*S*,*R*-**5**.

## Sample Availability

Samples of the compounds are available from the authors.
