# Supplementary material for: 3-(((1S,3S)-3-((R)-Hydroxy(4-(trifluoromethyl)phenyl)methyl)-4-oxocyclohexyl)methyl)pentane-2,4-dione: Design and Synthesis of New Stereopure Multi-Target Antidiabetic Agent
Source: Molecules. 2022 May 19;27(10):3265. doi: 10.3390/molecules27103265 (PMC9146474; doi:10.3390/molecules27103265)
Supplement: Supplementary file 1 [file molecules-27-03265-s001.zip › molecules-1696111-supplementary.pdf]

Supplementary Materials

3-(((1S,3S)-3-((R)-Hydroxy(4-(trifluoromethyl)phenyl)methyl)-4-oxocyclohexyl)methyl)pentane-2,4-dione: Design and Synthesis of New Stereopure Multi-Target Antidiabetic Agent

Abdul Sadiq [1,*,†], Mater H. Mahnashi [2,*,†], Umer Rashid [3], Muhammad Saeed Jan [4], Mohammed Abdulrahman Alshahrani [5] and Mohammed A. Huneif [6]

[1] Department of Pharmacy, Faculty of Biological Sciences, University of Malakand, Chakdara 18000, Dir (L), KP, Pakistan
[2] Department of Pharmaceutical Chemistry, College of Pharmacy, Najran University, Najran 55461, Saudi Arabia
[3] Department of Chemistry, COMSATS University Islamabad, Abbottabad Campus, Abbottabad 22060, Pakistan; umerrashid@cuiatd.edu.pk
[4] Department of Pharmacy, University of Swabi, Swabi 23561, KP, Pakistan; saeedjanpharmacist@gmail.com
[5] Department of Clinical Laboratory Sciences, Faculty of Applied Medical Sciences, Najran University,
P.O. Box 1988, Najran 61441, Saudi Arabia; maalshahrani@nu.edu.sa
[6] Pediatric Department, Medical College, Najran University, Najran 55461, Saudi Arabia; huneif@hotmail.com
* Correspondence: sadiquom@yahoo.com (A.S.); matermaha@gmail.com or mhmahneshi@nu.edu.sa (M.H.M.); ORCID: https://orcid.org/0000-0002-0154-7863 (A.S.); ORCID: https://orcid.org/0000-0002-4837-3653?lang=en (M.H.M.); Tel.: +92-(0)301-2297-102 (A.S.); +966-508-734-539 (M.H.M.)
† Current Address: Department of Pharmacy, University of Malakand, Chakdara 18000, Dir (L), KP, Pakistan

Figure S1. ^{1}H NMR of the purified compound (mixture of diastereomers).

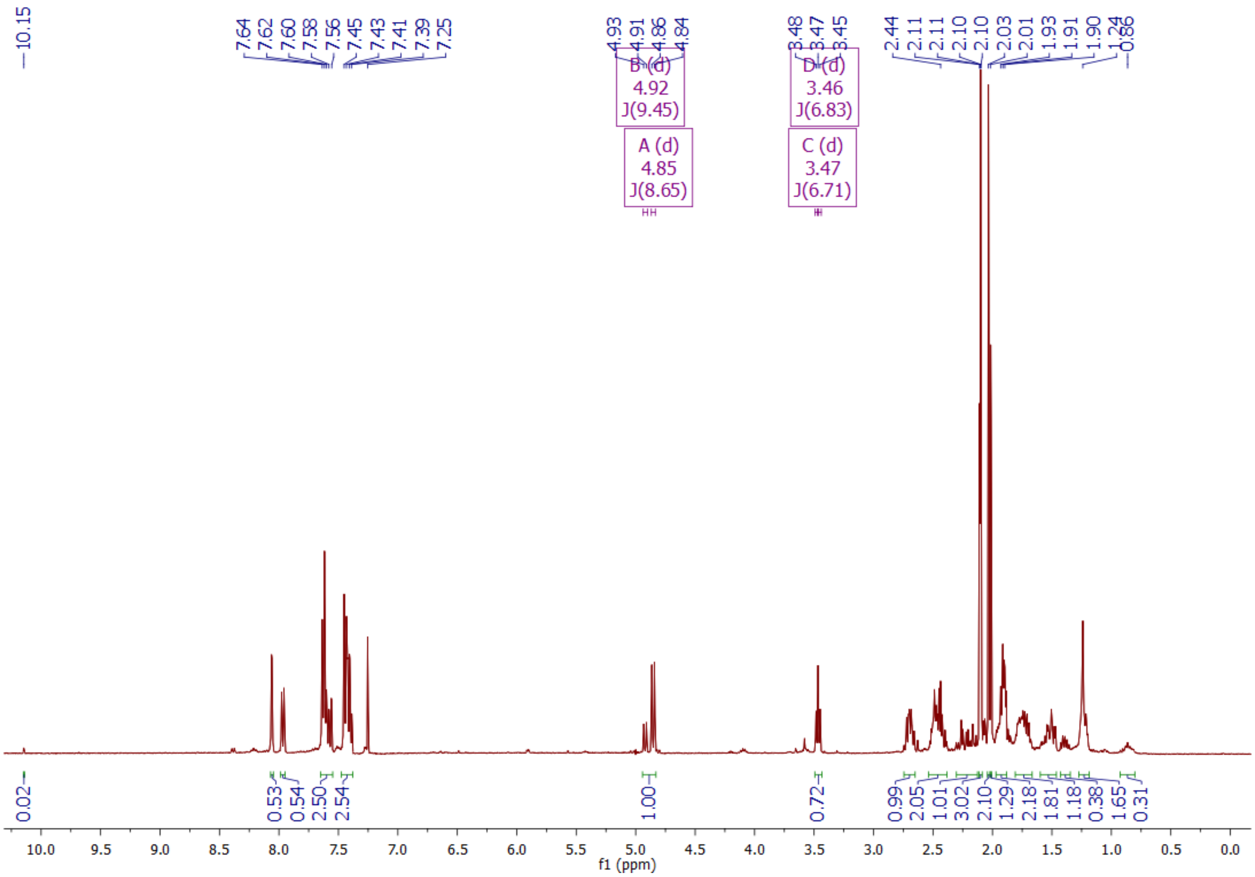

Figure S2. ^{13}C NMR of the purified compound (mixture of diastereomers).

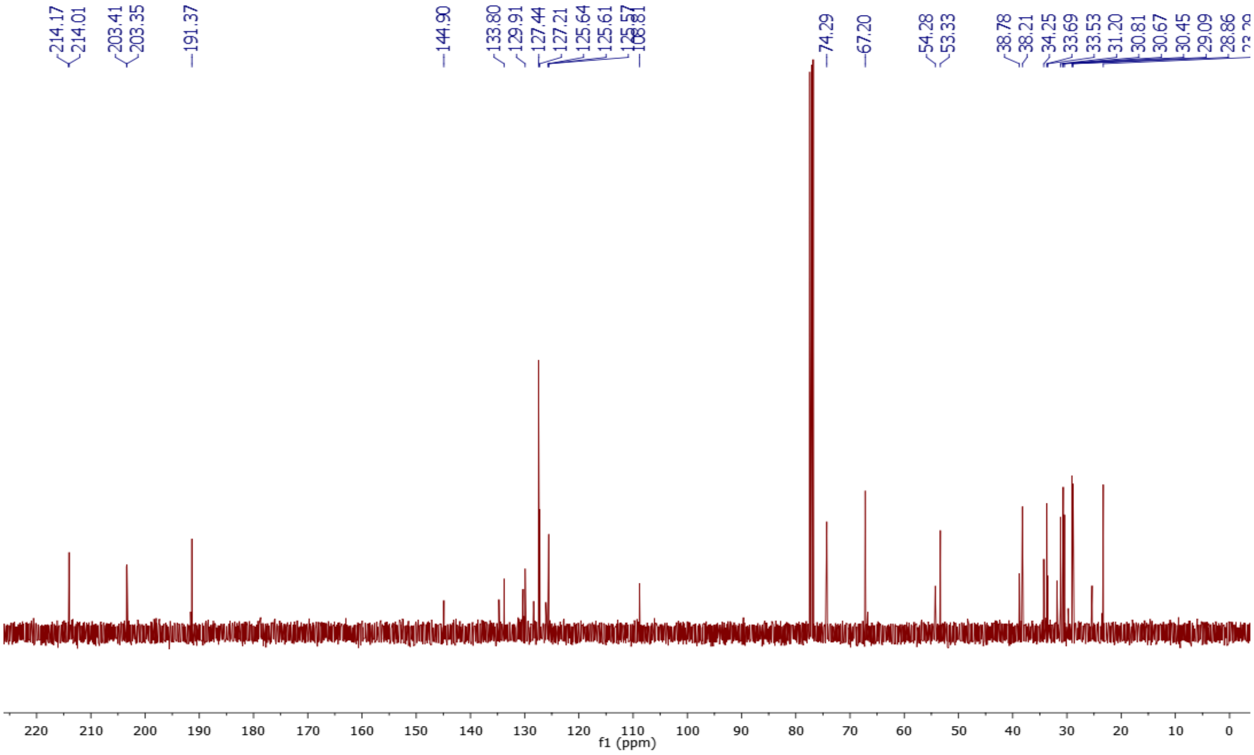

Figure S3. ^{1}H NMR of the stereopure compound *S*,*S*,*R*-**5**.

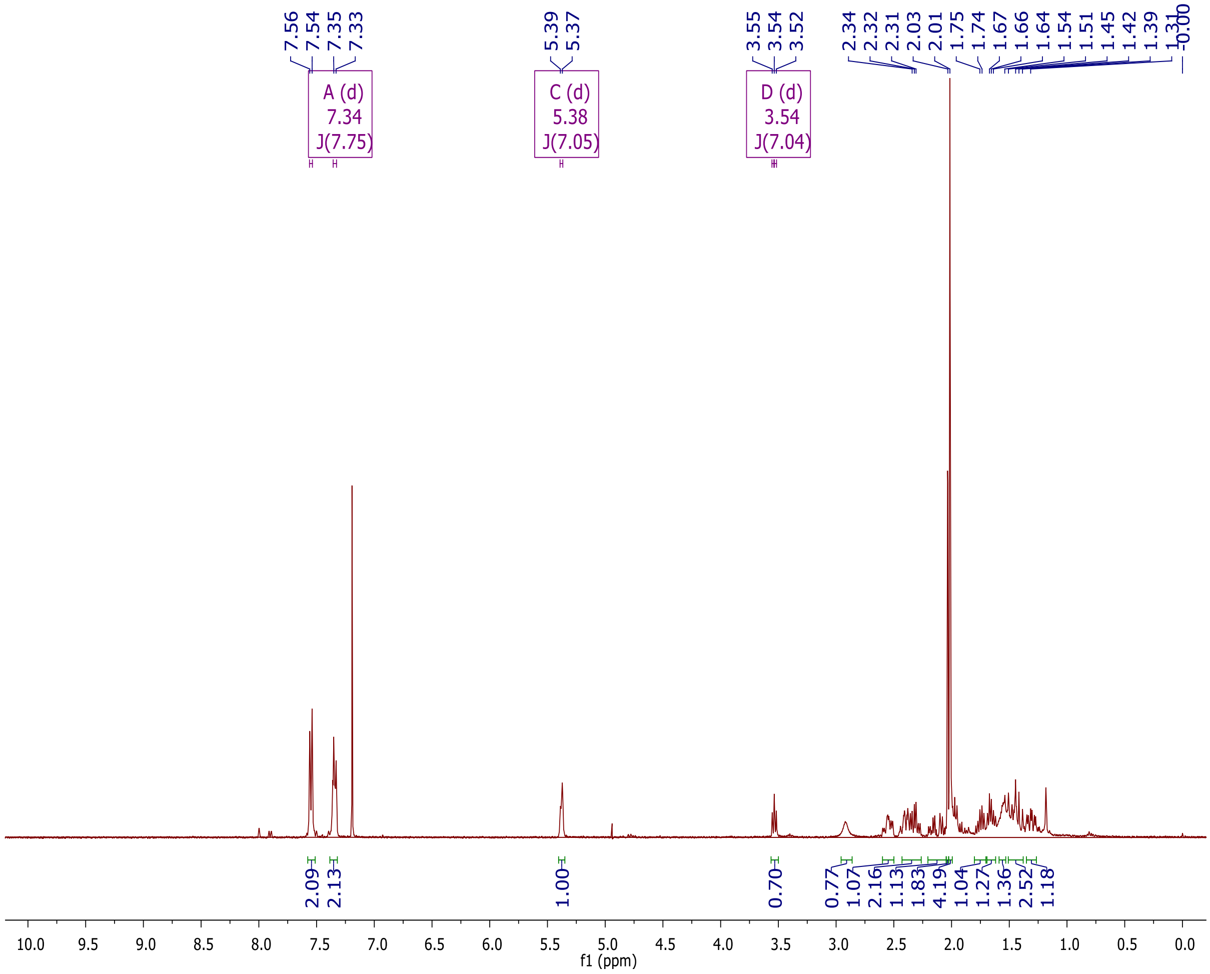

Figure S4. Expansions of the stereopure ¹H NMR of compound *S*,*S*,*R*-**5**.

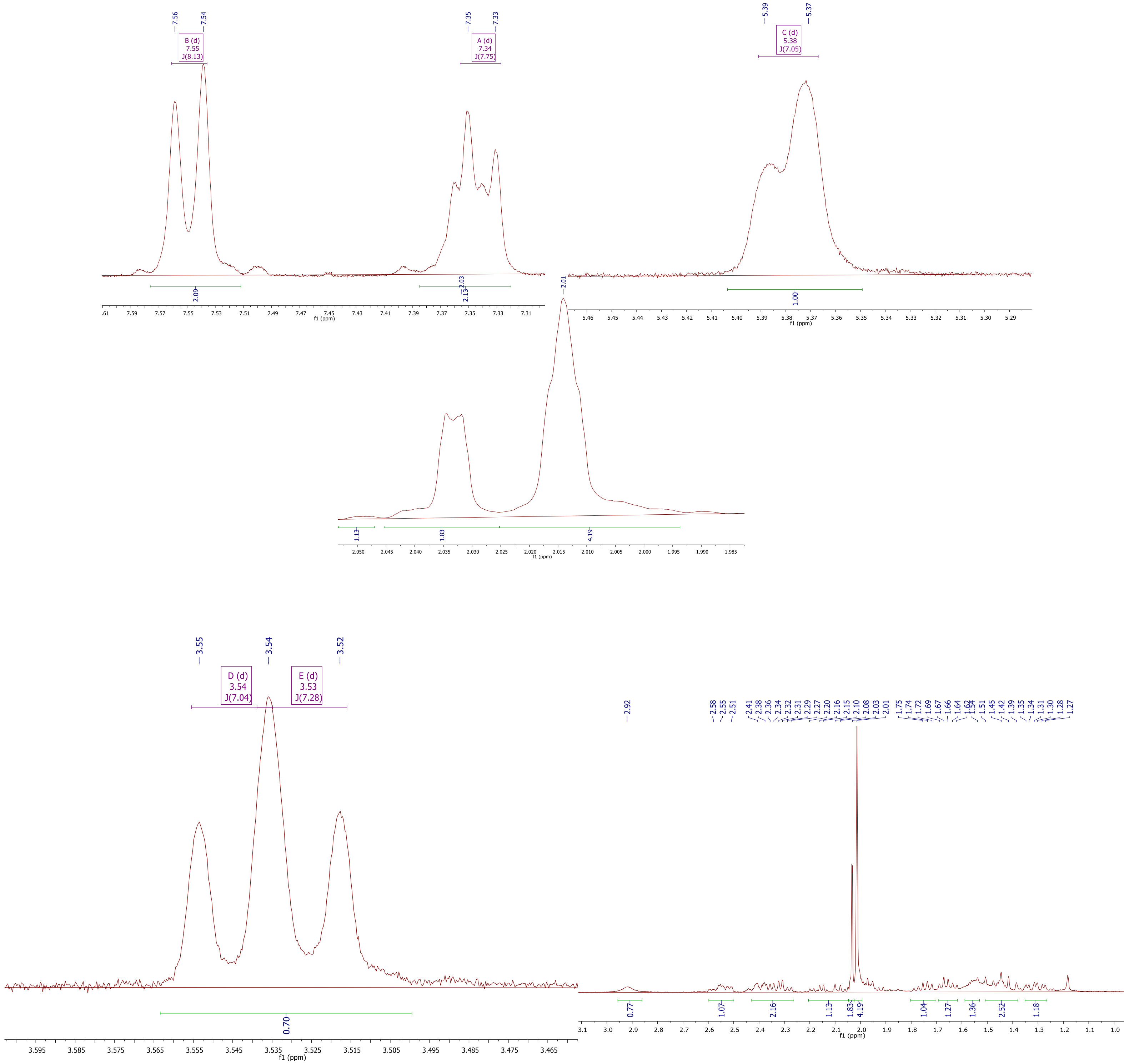

Figure S5. ^{13}C NMR of the stereopure compound *S*,*S*,*R*-**5**.

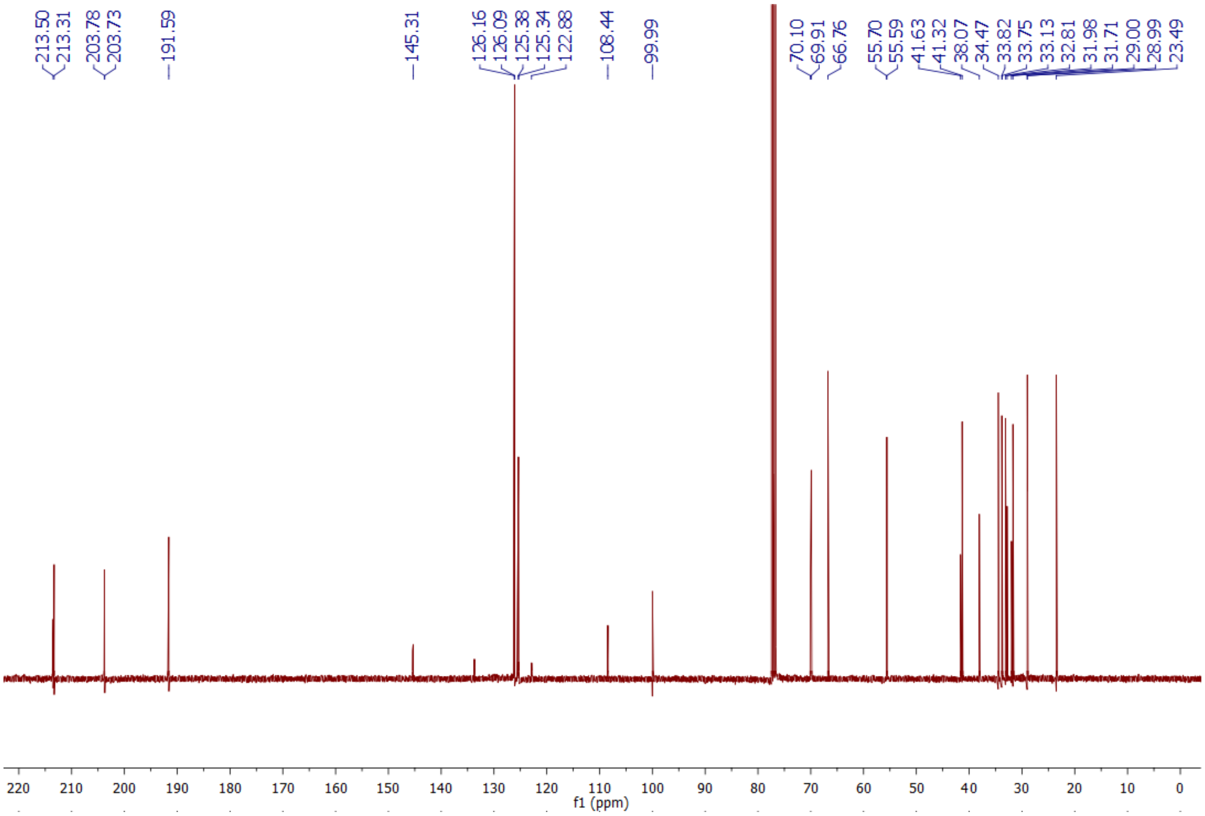

Figure S6. HRMS of the stereopure compound *S,S,R*-**5**.

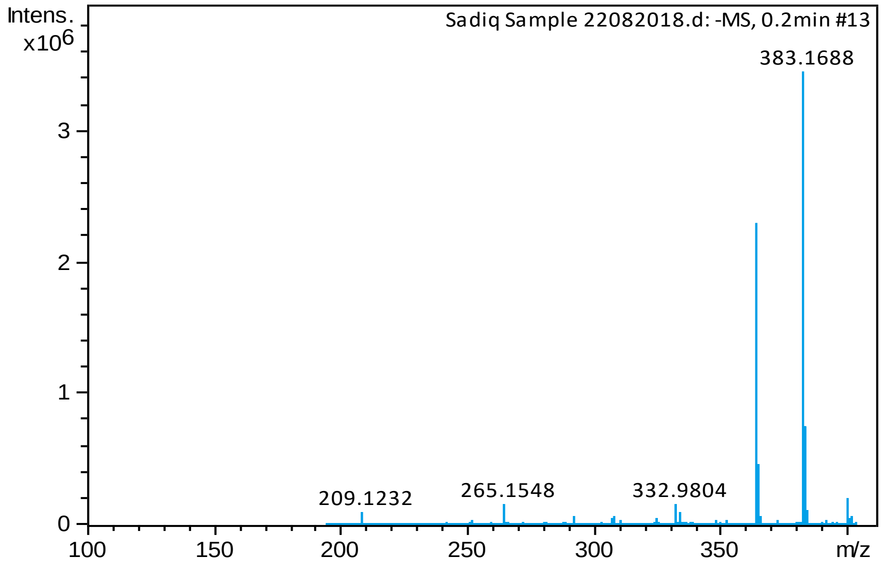

Figure S7. MS data of the stereopure compound *S*,*S*,*R*-**5**.

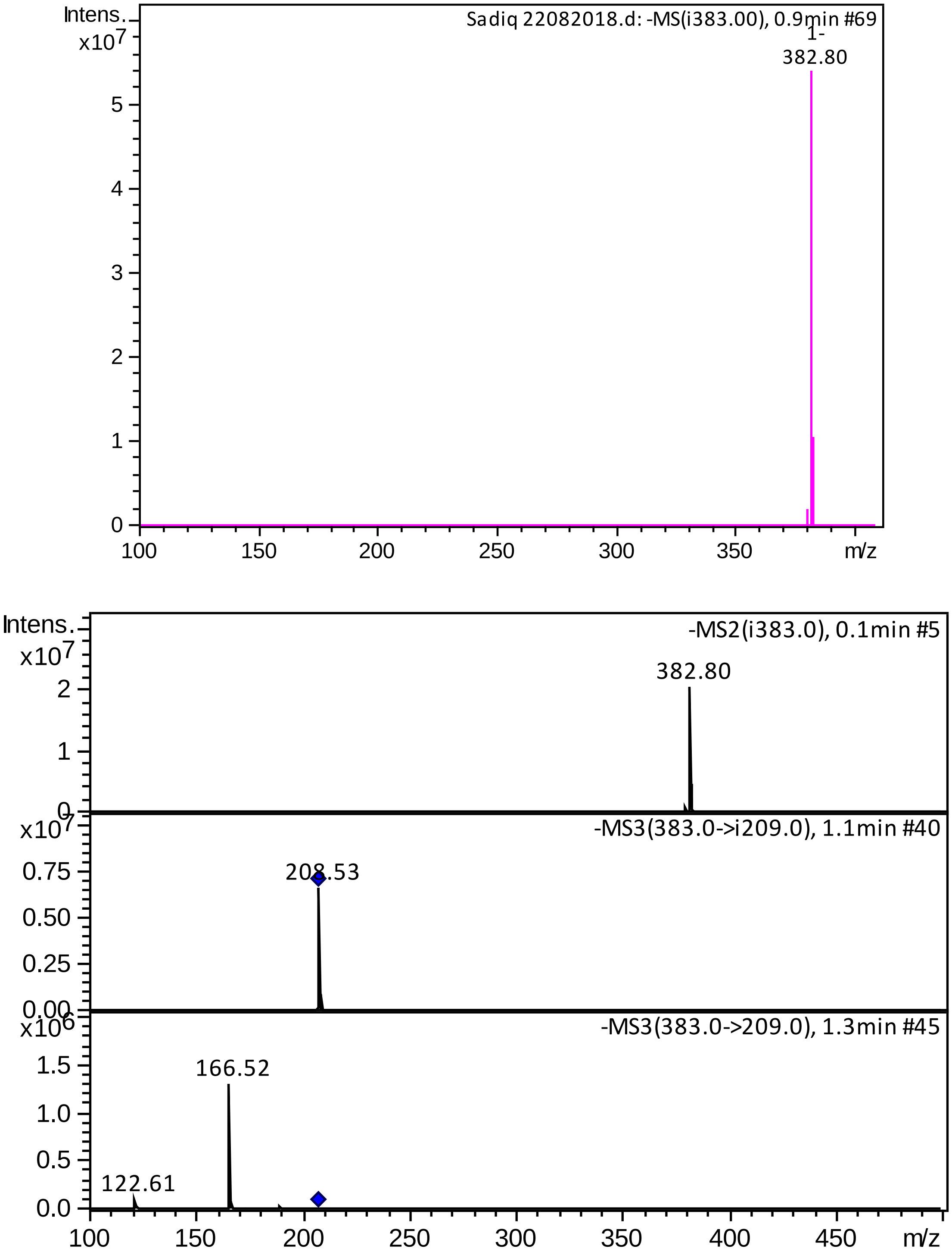

Figure S8. FT-IR analysis of the compound *S*,*S*,*R*-**5**.

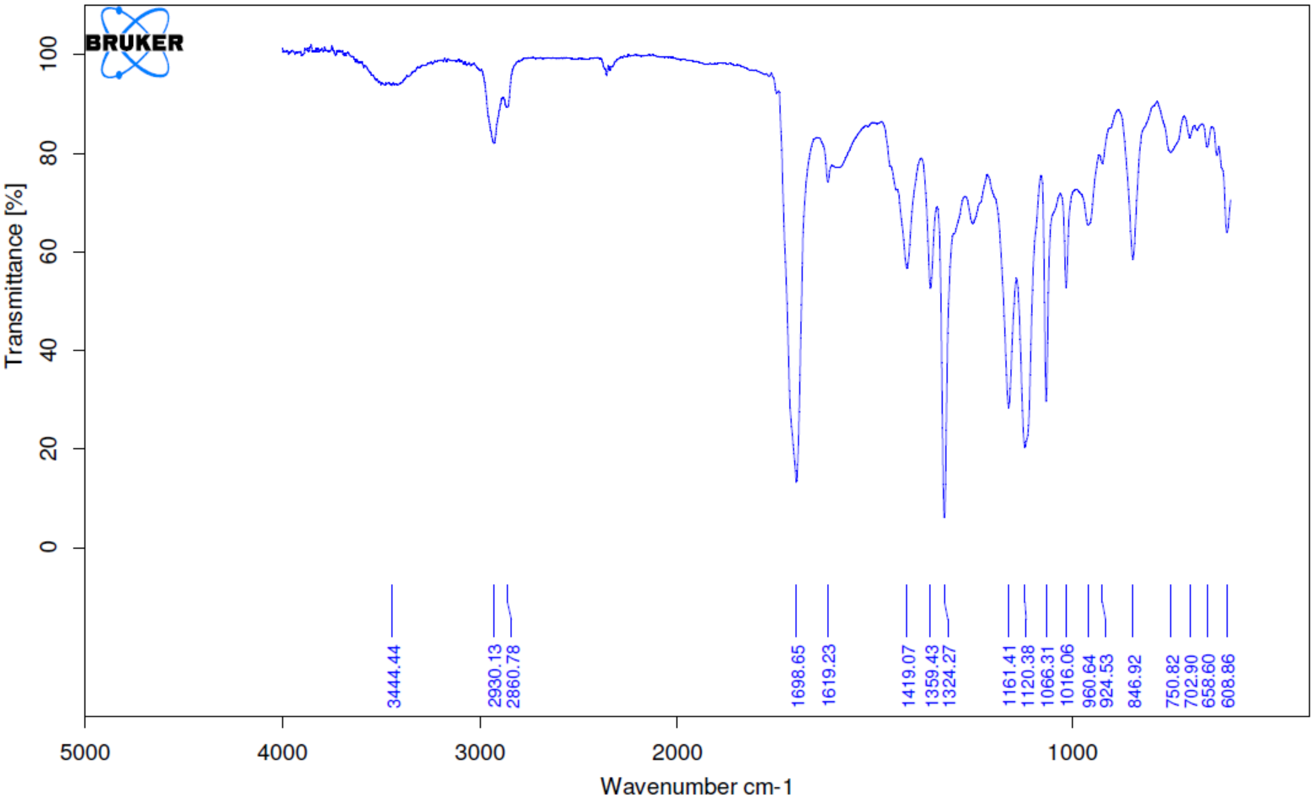

Figure S9. Chiral HPLC analysis of the racemic compound **Rac-5**.

==== Shimadzu LCsolution Analysis Report ====

Acquired by	: Admin
Sample Name	: SBKCF3 rac 14Nov
Sample ID	: SBKCF3 rac 14Nov
Vail #	: 1
Injection Volume	: 10 uL
Data File Name	: SBKCF3 rac 14Nov1.lcd
Method File Name	: 10 % 0.6 mL, 60 min.lcm
Batch File Name	:
Report File Name	: Musterreport.lcr
Data Acquired	: 14.11.2018 08:38:53
Data Processed	: 14.11.2018 09:18:58
Diese Probe heißt:	SBKCF3 rac 14Nov

<Chromatogram>

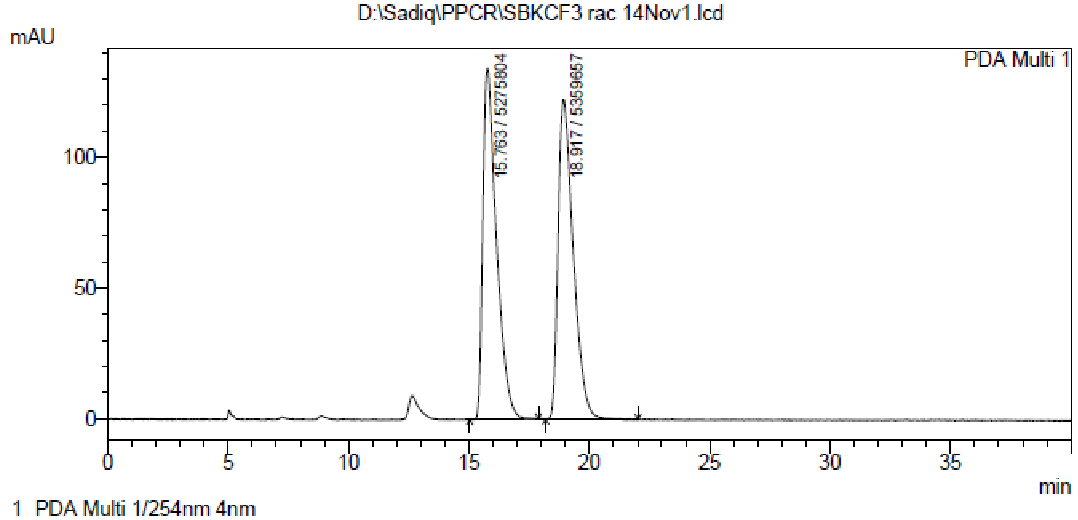

1 PDA Multi 1/254nm 4nm

<Results>

PDA Ch1 254nm 4nm

Peak #	Ret. Time	Area	Height	Area %	Height %	Width at 10% Height	Mark
1	15.763	5275804	134224	49.606	52.342	1.185	
2	18.917	5359657	122214	50.394	47.658	1.285	
Total		10635461	256438	100.000	100.000		

Figure S10. Chiral HPLC analysis of the stereopure compound *S*,*S*,*R*-**5**.

==== Shimadzu LCsolution Analysis Report ====

Acquired by	: Admin
Sample Name	: SBKCF3 before NMR
Sample ID	: SBKCF3 before NMR
Vail #	: 1
Injection Volume	: 10 uL
Data File Name	: SBKCF3 before NMR3.lcd
Method File Name	: 10 % 0.6 mL, 60 min.lcm
Batch File Name	:
Report File Name	: Musterreport.lcr
Data Acquired	: 14.11.2018 07:54:56
Data Processed	: 14.11.2018 08:35:01
Diese Probe heißt:	SBKCF3 before NMR

<Chromatogram>

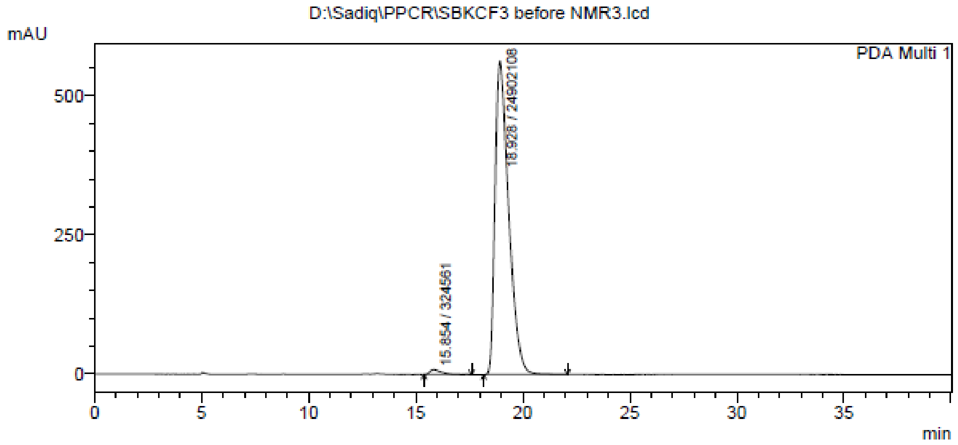

1 PDA Multi 1/254nm 4nm

<Results>

PDA Ch1 254nm 4nm

Peak #	Ret. Time	Area	Height	Area %	Height %	Width at 10% Height	Mark
1	15.854	324561	8754	1.287	1.531	1.131	
2	18.928	24902108	563170	98.713	98.469	1.299	
Total		25226669	571924	100.000	100.000		
